# Multi-level glyco-engineering techniques to generate IgG with defined Fc-glycans

**DOI:** 10.1038/srep36964

**Published:** 2016-11-22

**Authors:** Gillian Dekkers, Rosina Plomp, Carolien A. M. Koeleman, Remco Visser, Hans H. von Horsten, Volker Sandig, Theo Rispens, Manfred Wuhrer, Gestur Vidarsson

**Affiliations:** 1 Sanquin Research, Department Experimental Immunohematology, Amsterdam, The Netherlands, and Landsteiner Laboratory, Academic Medical Centre, University of Amsterdam, Amsterdam, The Netherlands; 2 Center for Proteomics and Metabolomics, Leiden University Medical Center, Leiden, The Netherlands; 3 ProBioGen AG, Berlin, Germany; 4 HTW-Berlin University of Applied Sciences, Life Science Engineering, Berlin, Germany; 5 Sanquin Research, Department Immunopathology, Amsterdam, The Netherlands, and Landsteiner Laboratory, Academic Medical Centre, University of Amsterdam, Amsterdam, The Netherlands

## Abstract

Immunoglobulin G (IgG) mediates its immune functions through complement and cellular IgG-Fc receptors (FcγR). IgG contains an evolutionary conserved *N*-linked glycan at position Asn297 in the Fc-domain. This glycan consists of variable levels of fucose, galactose, sialic acid, and bisecting *N*-acetylglucosamine (bisection). Of these variations, the lack of fucose strongly enhances binding to the human FcγRIII, a finding which is currently used to improve the efficacy of therapeutic monoclonal antibodies. The influence of the other glycan traits is largely unknown, mostly due to lack of glyco-engineering tools. We describe general methods to produce recombinant proteins of any desired glycoform in eukaryotic cells. Decoy substrates were used to decrease the level of fucosylation or galactosylation, glycosyltransferases were transiently overexpressed to enhance bisection, galactosylation and sialylation and *in vitro* sialylation was applied for enhanced sialylation. Combination of these techniques enable to systematically explore the biological effect of these glycosylation traits for IgG and other glycoproteins.

The fragment crystallizable (Fc) domain of immunoglobulin G (IgG) is recognized by IgG-Fc gamma receptors (FcγR) on myeloid and Natural-Killer (NK) cells, providing target recognition by, and activation of, immune effector cells^1^. A highly conserved glycan at position 297 in the Fc-region infers structural changes to the Fc-region required for binding to FcγR^2^,^3^,^4^. Subtle differences in the glycan composition at this site can affect the Fc-structure^5^,^6^,^7^, and may also alter the interaction with FcγR by direct contact^8^,^9^.

The Fc-glycan has a complex bi-antennary structure and is composed of *N*-acetylglucosamine (GlcNAc) and mannose groups. The core structure can be further extended with galactose, *N*-acetylneuraminic acid (sialic acid), fucose and bisecting GlcNAc (bisection) (Fig. 1a)^10^. The relative levels of all these glycoforms vary between individuals^10^. Furthermore, significant shifts in relative levels have been associated with age (all glycosylation features except fucosylation), infection and pregnancy^11^,^12^,^13^,^14^,^15^,^16^. The type of glycosylation is dependent on expression levels of glycosyltransferases and glycosidases in the secretion pathway of the antibody-producing plasma B cell^17^, except for sialylation which is probably also depended on extracellular sialyltransferase expression in the circulation^18^,^19^,^20^. The degree of fucosylation and bisection of IgG is generally very stable, with ~95% serum IgG being fucosylated and ~15% bisected. However, the number of galactoses which are present or absent on one or both arms of the bi-antennary glycan (reflected in G0, G1 and G2 structures) is much more variable: ~40% of the IgG antennae carry a galactose in healthy young individuals^10^. Sialic acid can be terminally added to the galactose, and is found on ~4% of IgG glycans^10^.

It has been known for several years that the absence of IgG-Fc core fucose vastly increases binding to FcγRIII^21^,^22^, a finding increasingly being used to improve the efficacy of therapeutic antibodies^23^,^24^,^25^. Although IgG produced in most human immune responses are highly fucosylated, we recently found that allogeneic immune responses against red blood cells and platelets can be skewed towards non-fucosylated IgG, the level of which correlates with disease outcome^14^,^15^,^26^,^27^. Similarly, immune responses with low-core fucosylation can also be found directed against HIV – particularly in elite controllers^28^.

Much less is known about the functionality of other glycosylation traits. Attachment of bisecting GlcNAc to the G0 glycan has been described to increased ADCC^29^. However, other studies have found that early overexpression of mannosyl (beta-1,4-)-glycoprotein beta-1,4-*N*-acetylglucosaminyltransferase (GNTIII) in the endoplasmic reticulum or early Golgi, not only results in increased IgG-bisection but also inhibits the incorporation of fucose, thereby increasing ADCC^30^,^31^. Thus, whether the bisecting GlcNAc directly affects IgG functionality has still not been adequately addressed. The function of the rest of the glycan features is less clear^14^,^16^,^32^,^33^, although a recent study indicates an additional role of galactose for FcγRIIIa binding^34^,^35^,^36^.

To study the effect of the different glycosylation traits, we established a glyco-engineering pipeline for manipulation of the IgG Fc-glycan. This glyco-engineering platform is simple and robust and takes place within human cells during production, harvesting the desired product within 5 days thereby eliminating the need for complex biochemical modifications. The methods are comprised of an array of approaches, including addition of (decoy) substrates of glycosyltransferases and transient overexpression of glycosyltransferases. These methods are not specific to HEK cells, and are in principle applicable to any eukaryotic cell-lines, providing tools to study the impact of glycans of any desired target protein.

Here we demonstrate the effectiveness of these glyco-engineering tools in HEK cells to manipulate specific glycosylation traits of the bi-antennary-Fc-glycan found in IgG without affecting other glycan traits or production levels. This collection of tools was aimed at decreasing the otherwise high fucosylation, increasing the normally low bisection, decreasing or increasing the normally intermediate galactosylation, and increasing the normally low sialylation. These platforms open up the possibility to combine these glyco-engineering tools to create at least 20 different glycoforms to explore their biological consequences.

## Results

### IgG produced in HEK Freestyle cells resembles plasma IgG

**Figure 1:**
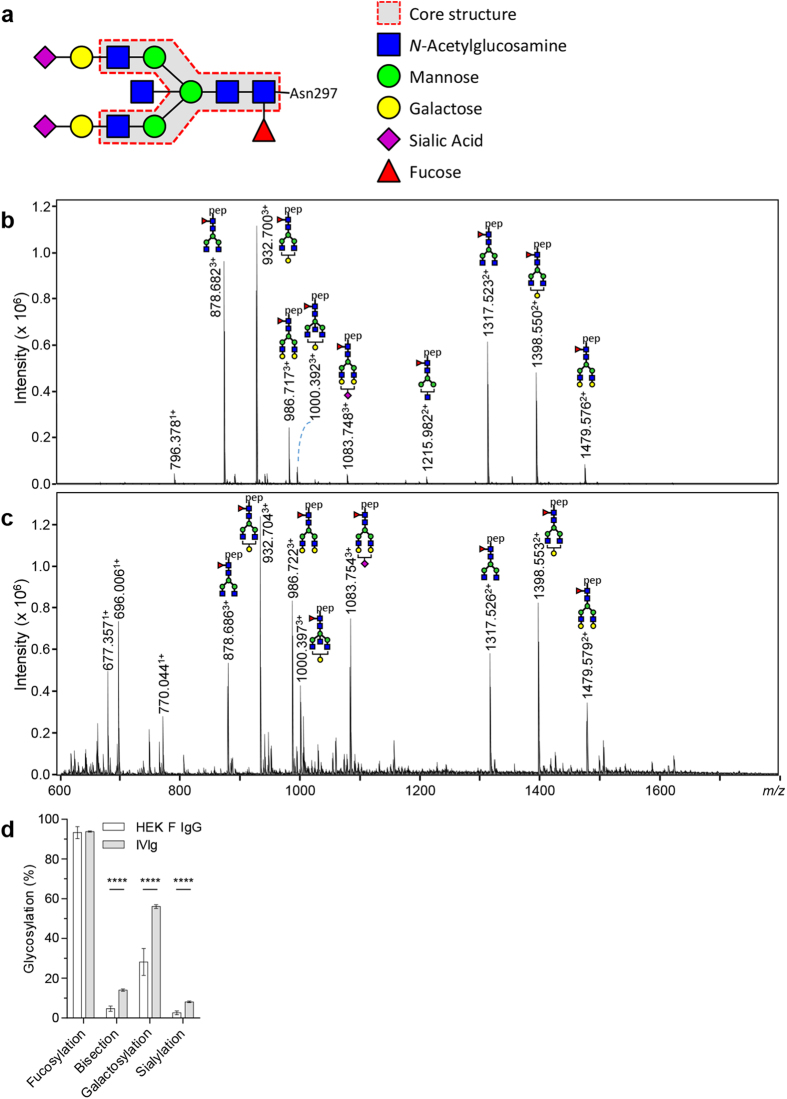
IgG1 from HEK freestyle produced IgG and IVIg have comparable glycoprofiles. (**a**) A schematic model of the bi-antennary glycan as present on IgG Fc at position N297. (**b**) NanoLC-ESI-MS spectrum of IgG1 produced in HEK cells and (**c**) human plasma-derived IVIg Kiovig IVIg (data obtained from ref. 37), exhibit a similar glycosylation pattern for the Fc glycan for each direct glycan trait. (**d**) The similarity is also evident when visualized as calculated derived glycan traits, IgG glycosylation profile of either HEK freestyle produced IgG (this study n = 28) or IVIg (n = 61, data obtained from ref. 37), Error bars indicate standard deviation (SD), **** denotes a statistical significance of p ≤ 0.0001 tested by unpaired t-test.

**Figure 2:**
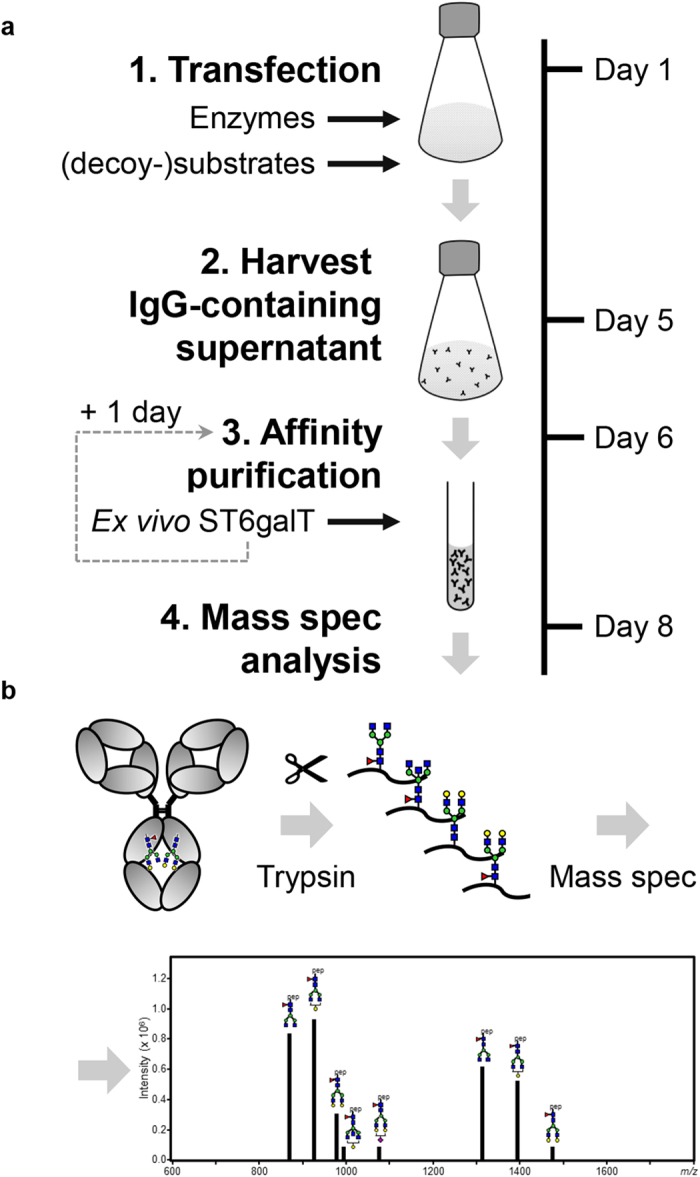
Overview of IgG production and intervention for glyco-engineering and glycosylation analysis. (**a**) Flowchart of IgG production in HEK freestyle cell line and glycan analysis by mass spectrometry, illustrating the 4 steps of IgG production on a time scale, with arrows indicating the glyco-engineering methods employed at each step. After the *in vitro* ST6GALT treatment, an additional purification step was required (dashed line with arrow). (**b**) To analyse the glycosylation profile, the resulting IgG was digested with trypsin and the glycopeptides encompassing the *N*-glycan were analysed by mass spectrometry.

In order to recapitulate the variability of the conserved N297-glycan in the Fc-region of human IgG (Fig. 1a), we first tested if the glycosylation of IgG produced in Human-Embryonic Kidney (HEK) cells resembles that of normal human plasma-derived IgG. After trypsinization of the purified IgG, the resulting glycopeptides encompassing the *N*-linked glycosylation site at position 297 were analysed by mass spectrometry (Fig. 1b–d). The obtained glycosylation patterns were similar to those found in a pool of IgG derived from plasma of thousands of plasma donors (IVIg) (Fig. 1d)^37^. In general, plasma and HEK-derived IgG were highly fucosylated, with roughly half of the glycans galactosylated, with low levels of sialylation and bisecting *N*-acetylglucosamine (bisection). To change the composition of the complex bi-antennary Fc-glycan, we therefore focused our glyco-engineering efforts on the generation of several tools aimed at decreasing fucosylation, increasing bisection, decreasing or increasing galactosylation, and increasing sialylation. These tools and methods are described in Fig. 2.

### Reducing core-fucosylation

**Figure 3:**
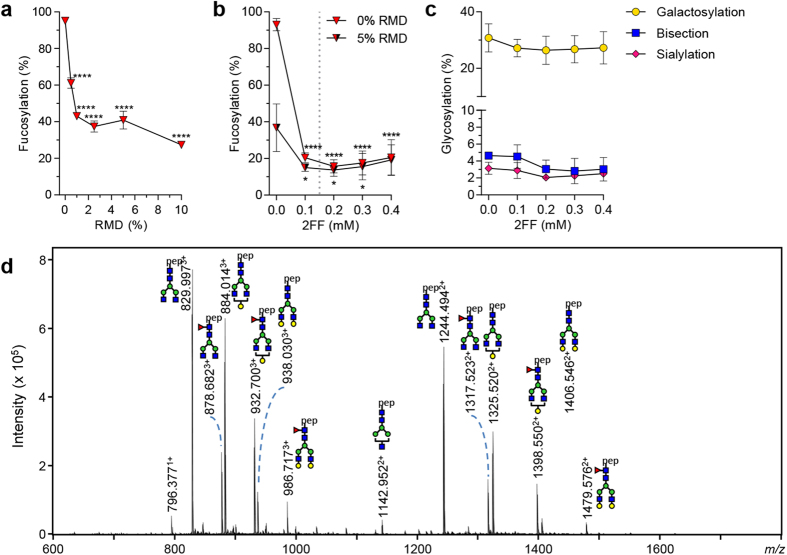
Decreasing fucosylation. (**a**) The fucose level of IgG1 N297, produced by transfection of IgG heavy and light chain vector in combination with co-transfection of RMD vector. (**b**) or with addition of 2FF or a combination of 2FF and co-transfection with 5% RMD. (**c**) Effect of 2FF addition on other derived glycosylation traits (bisection, galactosylation, sialylation). (**d**) NanoLC-ESI-MS spectrum of IgG produced with 0.4 mM 2FF. The data represents means and SD of three combined independent experiments, * and **** denote a statistical significance of p ≤ 0.05 and p ≤ 0.0001, respectively, as tested by one-way ANOVA using Dunnett’s multiple comparisons test comparing untreated cells with treated. The vertical dotted lines in (**b**,**c**) represent the designated optimal concentrations of 2FF.

We first tested the potential of the bacterial enzyme GDP-6-deoxy-D-lyxo-4-hexulose reductase (RMD)^38^ to block the *de novo* production of fucose in HEK cells. Co-transfection of RMD together with IgG1 indeed resulted in decreased incorporation of core fucose in the N297-Fc glycan, from 94% to 27% (Fig. 3a). An alternative method to decrease fucosylation in IgG is to use the decoy substrate 2-deoxy-2-fluoro-l-fucose (2FF)^14^,^39^. Addition of 2FF to the culture media resulted in reduced incorporation of fucose in the IgG-Fc glycan down to 15% (Fig. 3b). Further titration showed that 0.15 mM is the optimal 2FF concentration and that time of 2FF addition does not influence 2FF efficacy (Fig. S1). Combining both RMD and 2FF resulted in no further reduction beyond that seen with 2FF alone (Fig. 3b). 2FF treatment did not affect the level of galactosylation, sialylation, bisection (Fig. 3c), or IgG yield (Fig. S2b). Neither did it affect high-mannose and hybrid-type glycosylation (Fig. S3a). These glycoforms are also found on IgG in serum but in very low amounts (1–2.5%)^40^,^41^, as well as in IgG resulting from monoclonal antibody production. Even at concentrations above the optimum of 0.15 mM (Fig. 3a,b), 2FF addition resulted in IgG1 with the most abundant glycan forms consisting of afucosylated species (Fig. 3d).

### Increasing GlcNAc (bisection)

**Figure 4:**
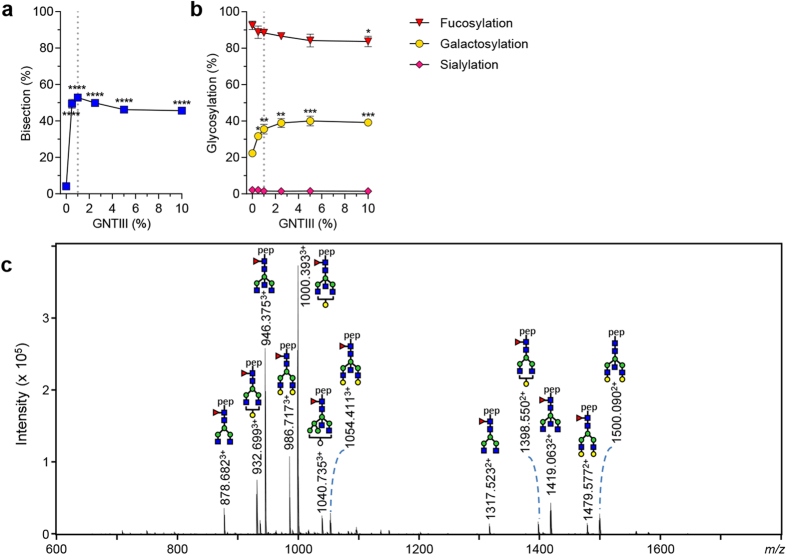
Increasing bisecting GlcNAc. (**a**) The level of bisecting GlcNAc (bisection) on IgG1 N297 after co-transfection of IgG heavy and light chain vectors with GNTIII vector. (**b**) The effect of GNTIII co-transfection on other derived glycosylation traits (fucosylation, galactosylation, sialylation). (**c**) NanoLC-ESI-MS spectrum of IgG produced with 1% GNTIII co-transfection. The data represents means and SD from two independent experiments carried out in an identical fashion, representative of 3 independent experiments *, **, *** and **** denote a statistical significance of p ≤ 0.05, p ≤ 0.01, p ≤ 0.001 and p ≤ 0.0001, respectively, as tested by one-way ANOVA using Dunnett’s multiple comparisons test comparing untreated cells with treated. The vertical dotted lines in (**a**,**b**) represent the designated optimal concentrations of GNTIII.

The human GlcNAc transferase III, beta-1,4-mannosyl-glycoprotein 4-beta-*N*-acetylglucosaminyltransferase (GNTIII) enzyme is known to be responsible for the addition of GlcNAc to complex bi-antennary glycans^30^. Addition of 1% DNA encoding for GNTIII to the transfection mixture resulted in maximally 53% incorporation of bisecting GlcNAc in the IgG-Fc (Fig. 4a). The co-transfection of GNTIII resulted in a slight increase in galactosylation (from 22 to 36% at the optimum of 1% concentration of GNTIII, Fig. 4a,b) and decrease in fucosylation from 93 to 88% (Fig. 4b) without any loss in antibody yield (Fig. S2c). A slight, although not significant, increase in hybrid-type and high-mannose glycoforms (from 5 to 7% of total glycoforms) was observed upon this treatment (Fig. S3b). The most abundant glycoforms of IgG1 produced were bisected IgG1-glycoforms (Fig. 4c).

### Decreasing galactosylation

**Figure 5:**
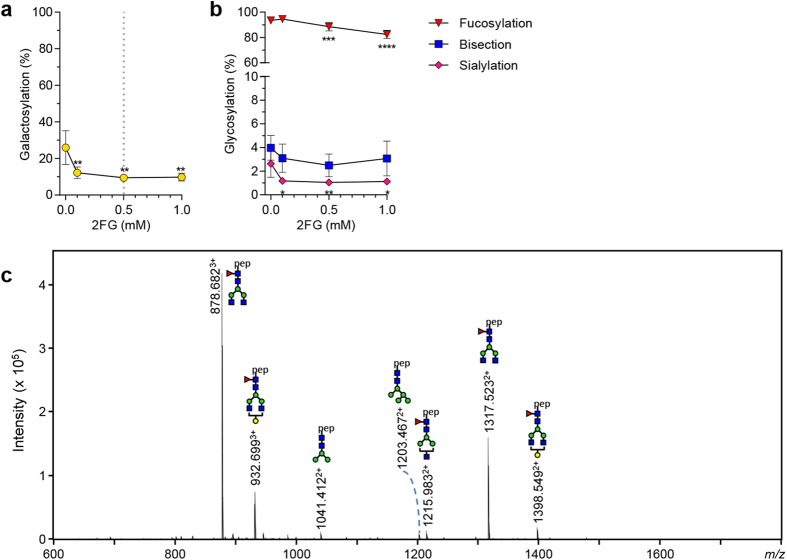
Decreasing galactosylation. (**a**) The galactosylation level of IgG1 N297 produced with addition of 2FG. (**b**) Effect of 2FG addition on other derived glycosylation traits (fucosylation, bisection, sialylation). (**c**) NanoLC-ESI-MS spectrum of IgG produced with 1 mM 2FG. The data represents means and SD of four combined independent experiments, *, **, *** and **** denote a statistical significance of p ≤ 0.05, p ≤ 0.01, p ≤ 0.001 and p ≤ 0.0001, respectively, as tested by one-way ANOVA using Dunnett’s multiple comparisons test comparing untreated cells with treated. The vertical dotted lines in (**a**,**b**) represent the designated optimal concentrations of 2FG.

To reduce the level of galactosylation of HEK-produced IgG, we identified the galactose analogue 2-deoxy-2-fluoro-d-galactose (2FG) as a novel and specific blocker of galactosylation (Fig. 5a). By addition of 0.5 mM 2FG to the culture medium, incorporation of galactose into the IgG1 N297-Fc glycan was reduced down to 9%. A slight but significant drop in fucosylation (from 93% to 89%) and an expected significant drop in sialylation (from 2.6% to 1.0%) (Fig. 5b), as well as a slight elevation of high-mannose and hybrid-type glycans was observed (Fig. S3c) (from 4 to 13%, 9% at the optimum of 0.5 mM 2FG (Fig. 5a)). This method resulted in almost exclusively IgG1 without galactose (Fig. 5c). Addition of a higher concentration of 2FG decreased IgG-yields (Fig. S2d) without further reduction of galactosylation (Fig. 5a).

### Increasing galactosylation

**Figure 6:**
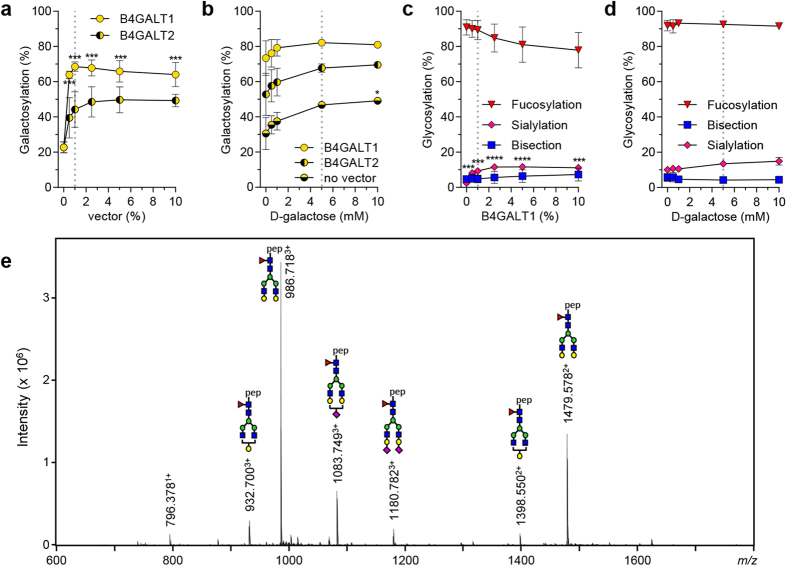
Increasing galactosylation. (**a**) The galactosylation level of IgG1 N297 after co-transfection of IgG heavy and light chain vectors with B4GALT1 or B4GALT2 vectors, and (**b**) after addition of D-galactose or in combination with co-transfected 1% B4GALT1, or 1% B4GALT2. (**c**) The effect of B4GALT1 co-transfection or (**d**) or D-galactose addition on the other derived glycosylation traits (fucosylation, bisection, sialylation). (**e**) NanoLC-ESI-MS spectrum of IgG produced with 1% B4GALT1 co-transfection and 1 mM D-galactose addition. The data represents means and SD from two independent experiments carried out in an identical fashion, representative of 3 independent experiments, *, *** and **** denote a statistical significance of p ≤ 0.05, p ≤ 0.001 and p ≤ 0.0001, respectively, as tested by one-way ANOVA using Dunnett’s multiple comparisons test comparing untreated cells with treated. The vertical dotted lines in (**a**,**b**) represent the designated optimal concentrations of B4GALT1, and in (**c**,**d**) the optimal concentrations of D-Galactose.

*In vivo*, galactose can be added to *N*-linked glycans by several different galactosyl transferases. In B-cells β-1,4-galactosyltransferase 1 (B4GALT1) is expressed^17^, with β-1,4-galactosyltransferase 2 (B4GALT2) having very similar if not identical substrate preference. When comparing both B4GALT1 and B4GALT2 for inducing galactosylation we found that inclusion of 1% DNA encoding for B4GALT1 to the transfection mix induced galactosylation up to 70%, outperforming B4GALT2 which reaches 50% galactosylation (Fig. 6a). We noted that higher dosages than the optimum of either 1% B4GALT1 or B4GALT2 dramatically reduced the yield of IgG production (Fig. S2e).

In addition to increasing the level of IgG1-Fc galactosylation through enzyme manipulation, we also tested the effect of substrate availability on galactosylation. By adding D-Galactose to the medium, a gradual increase in galactosylation was observed, which was further increased by co-transfection of galactosyl transferases (Fig. 6b). Co-treatment of B4GALT1 with D-Galactose did not adversely affect production levels (Fig. S2F). We observed a slight, expected increase in sialylation (Fig. 6c), and unexpected increases of high-mannose and hybrid-type glycans when increasing galactosylation by increasing the level of B4GALT1 (from 2 to 23%, 12% at the optimum of 1% B4GALT1 Fig. S3d). However, combining 1% B4GALT1 and D-Galactose did not significantly increase high-mannose structures (Fig. S3e). An optimum was reached by co-transfection of 1% B4GALT1 and 5 mM D-galactose addition, resulting in 82% galactosylation (Fig. 6e).

### Increasing sialylation

**Figure 7:**
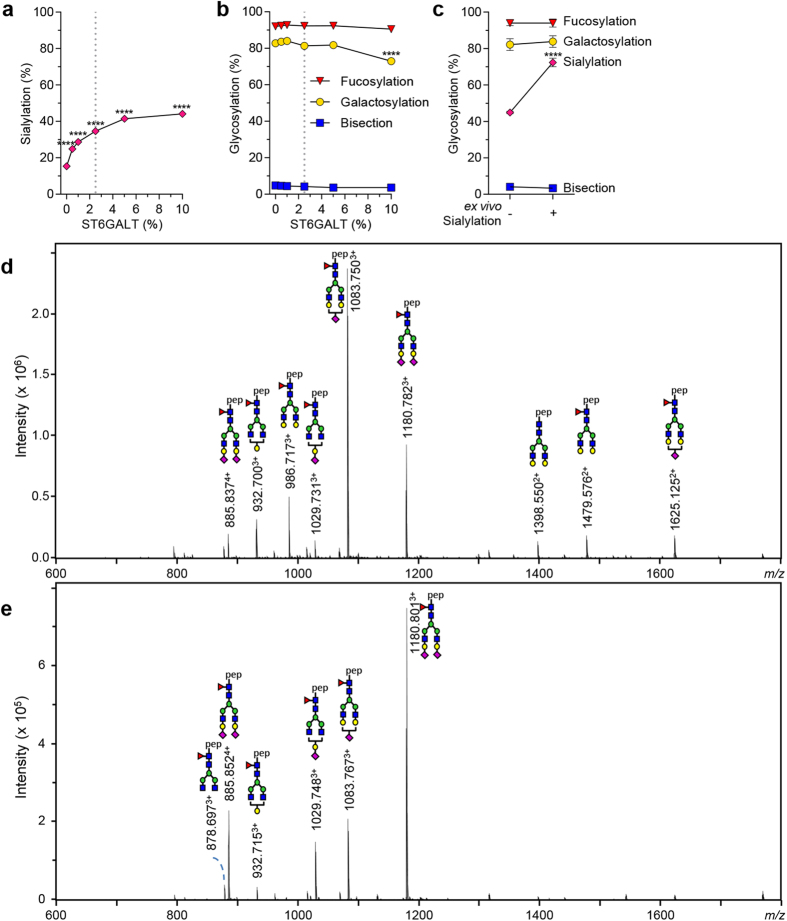
Increasing sialylation. (**a**) The sialylation level of IgG1 N297 after co-transfection of IgG heavy and light chain vectors with 1% B4GALT1 and addition of 5 mM D-galactose to increase galactosylation, in combination with increasing amount of co-transfected ST6GALT. N = 2 (**b**) Effect of ST6GALT co-transfection on other derived glycosylation traits. N = 2 (**c**) *In vitro* sialylation by ST6GALT of highly galactosylated and sialylated IgG1. Before treatment N = 3, after treatment N = 7 (**d**) NanoLC-ESI-MS spectrum of IgG produced with the identified optimal co-transfection additions of B4GALT1 (1%) and ST6GALT (2,5%) with the addition 1 mM D-galactose, and (**e**) spectrum of *in vitro* sialylated IgG produced in (**d**). The data represents means and SD, **** denotes a statistical significance of p ≤ 0.0001 tested by one-way ANOVA using Dunnett’s multiple comparisons test (**a**,**b**) or unpaired t-test (**c**) comparing untreated cells or IgG respectively with treated. The vertical dotted lines in (**a,b**) represent the designated optimal concentrations of ST6GAL.

As sialic acid is the terminal sugar group on the complex glycan (Fig. 1a), a high level of galactosylation is needed to increase the level of sialylation. The optimal tools for the addition of galactose (B4GALT1 co-transfection and D-galactose addition, Fig. 6) were combined with co-transfection of β-galactoside alpha-2,6-sialyltransferase 1 (ST6GALT), providing the α-2,6-linkage found in human IgG^42^. Sialylation levels gradually increased with increasing the amounts of ST6GALT in the co-transfection mix (Fig. 7a). Higher levels of ST6GALT vector affected IgG production levels negatively (Fig. S2g) and also slightly affected galactosylation levels while leaving fucosylation, bisection, high-mannose and hybrid-types unchanged (Fig. 7b, and Fig. S3f). To further increase sialylation of the IgG, the highly galactosylated and sialylated IgG was treated *in vitro* with recombinantly produced ST6GALT and cytidine-5′-monophospho-*N*-acetylneuraminic acid (CMP-NANA) substrate, which resulted in 74% sialylation (Fig. 7c). By transfection only, optimal ST6GALT concentration was determined to be 2.5% vector relative to antibody vector, reaching 44% sialylation without affecting either production (Fig. S2g) or galactosylation level (Fig. 7b). By this treatment roughly half of the available galactose residues were sialylated, with both mono- and di-sialylated glycan species present in similar amounts (Fig. 7d). By *in vitro* sialylation, the level of di-sialylated species was further increased, with roughly 90% of available galactose residues covered (Fig. 7e).

## Discussion

Antibody Fc glycosylation is important due to the differential influence of certain glycoforms on the effector functions, but the impact of all the different glycosylation patterns is unknown. In the present study glyco-engineering tools were developed to produce IgG, representing all common Fc-glycoforms, but also more extreme yet natural glycoforms.

To achieve this, the glycosylation machinery was adapted by co-transfecting different glycosyltransferases. Additionally, the already existing machinery was blocked using novel decoy-substrates, or enhanced by supplying natural substrates. This system is completely serum-free and human, and thus devoid of non-human cell glycan-additions found in Chinese Hamster Ovary (CHO) or mouse myelomas^43^,^44^, as for example the α-2,3-linked neuraminic acid and alpha gal (Galα1–3Gal).

The tools we describe target all the variable glycan traits normally found on the bi-antennary glycan of human IgG. These include bisecting GlcNAc (normally very low), fucosylation (very high) galactosylation (intermediate or low) and sialylation (very low). The incorporation of fucose was efficiently blocked by adding 2FF as previously described^14^,^39^, or by co-transfecting the enzyme RMD^38^. Of these two, the 2FF-mediated fucosylation decrease was not further reduced by RMD. An explanation for the RMD vector not being optimally effective could be that this vector was not codon optimized for expression in human cells but for CHO cells, but the apparent plateau reached argues against this. We also cannot exclude the presence of residual fucose in the medium which may explain why 0% fucosylation is not reached with either the RMD or the 2FF method. We could effectively inhibit the incorporation of galactosylation by the addition of 2FG, a compound previously described to block *N*-glycosylation^45^. However, we found this not to affect *N*-linked glycosylation but to specifically affect incorporation of galactose. In contrast, increased galactosylation was achieved by co-transfection of B4GALT1, and a further increase was brought about by inclusion of D-galactose substrate in the medium. Furthermore, we enhanced sialylation by co-transfection of ST6GALT, but could only reach approximately 50% of the theoretical maximum determined by the level of galactosylation. Co-transfection with higher amounts of ST6GALT vector resulted in minor elevation of IgG-sialylation, also described previously for CHO cells^44^ but negatively affected IgG yields and galactosylation.

The reason for the low efficacy for incorporation of sialic acid is unclear but may relate to the enclosed Fc-cavity, where the glycans are located, which may not allow for complete access of the enzymes involved in either addition of galactose and sialic acid. However, studies comparing free and Fc-glycans only show a factor 5 or so less accessibility of ST6GAL1 to the Fc-297-attached glycan^46^,^47^. The time given for the glycosylation machinery to manipulate the glycan may also be too short for full sialylation. Circumventing this with a limiting concentration of Brefeldin A, stalling protein secretion, did however not enhance sialylation, but resulted in strong elevation of high-mannose and hybrid-type glycans (not shown). Addition of the sialic acid substrate *N*-acetylmannosamine (ManNAc) or acetylated ManNAc, previously shown to be taken up by cells^48^,^49^, did not elevate sialylation levels (not shown), suggesting that substrate availability was not limited for ST6GALT. However, by *in vitro* sialylation, described also previously by Anthony *et al*.^42^, we were able to increase sialylation to levels approaching maximum possible allowed by the already incorporated galactose.

Most tools were highly specific, affecting only the targeted glycan trait. However, co-transfection of GNTIII not only resulted in an increase in bisecting GlcNAc but also in a slight but significant increase in galactosylation. Addition of 2FG also resulted in a slight decrease of fucosylation and a slight enhancement of high-mannose and hybrid-type glycans. Recently, it has been demonstrated that decorations of the IgG Fc N-glycan core affect the accessibility and consequently processing of Fc N-glycan antennae^50^. We speculate that the increased galactosylation observed with expression of the bisecting enzyme, GNTIII, as observed in our study, may be caused by the conformational change induced by bisection. Alternatively, changing the expression of certain glycosyltransferases and/or increasing the availability of substrates will also affect glycosylation of other proteins, even glycosyltransferases themselves, that may affect their activity. Higher doses of this substrate also negatively affected cell viability and IgG yields. As expected, enhancing galactosylation also increased sialylation. This effect was minor, but was observed for both B4GALT1 and B4GALT2, the former being expressed in B cells and being more active. Enhancement of galactosylation with- and without further enhancement of sialylation levels resulted in a slight increase of high-mannose and hybrid-type glycan species (<12% for final conditions chosen).

We believe the methods described here greatly improve upon previous attempts of glycan-engineering of potential therapeutic proteins in antibody-producing cells themselves. Furthermore, these methods require little or no expensive *in vitro* enzymatic manipulation of the desired protein, or complex chemical modifications. Due to the general nature of the methods, these can also be implemented in other cell lines producing any desired *N*-linked glycoprotein or even viral particles. The feasibility of implementing these methods acting on the producer cell line is higher than post-production enzyme or chemical modifications which also require additional purification steps. Finally, the levels of all glycan additions can all be fine-tuned to achieve a desired level, which may not be possible by genetic knockdown or stable transfection of glycosyltransferases or glycosidases. In the near future we are planning on combining all these tools to address the collective effects of changing two to four of the common glycan adducts on binding to FcγR, complement and their functional properties.

## Materials and Methods

### Strains and reagents

*Escherichia coli* strain DH5α was used for recombinant DNA work. Restriction endonucleases and DNA modification enzymes were obtained from Thermo Fisher Scientific (Life Technologies, Waltham, Massachusetts, USA). Oligonucleotides were obtained from Geneart (Life Technologies) or Integrated DNA Technologies (Coralville, Iowa).

### Expression vector constructs

A single-gene vector anti-TNP IgG1 heavy- and kappa-light chain encoding sequences were cloned as described previously by Kruijssen *et al*.^51^ into a pEE14.4 (Lonza) expression vector. In brief, the codon-optimized V gene for both heavy and light chain, including 5′-HindIII and 3′-NheI or 5′-HindIII and 3′-XhoI restriction sites respectively, Kozak sequence, and HAVT20-leader sequence^52^, were designed and ordered from Geneart (Life Technologies). The HindIII-NheI or HindIII-XhoI fragments for the codon-optimized heavy or light chain was ligated into γ or κ constant region flanking 3′-EcoRI restriction site respectively. The HindIII-EcoRI fragment for the codon-optimized light chain was ligated into pEE14.4 (Lonza), and the HindIII-EcoRI fragment for the heavy chain was ligated into pEE6.4 (Lonza). A single-gene vector encoding IgG1 was subsequently generated by ligation of the BamHI-NotI fragment from pEE6.4 [including a cytomegalovirus (CMV) promoter], IgG1 heavy chain, and poly(A) into the light-chain-encoding pEE14.4 vector.

Protein sequences of human enzymes B4GALT1 (NCBI reference sequence NP_001488.2), B4GALT2 (NP_001005417.1), ST6GALT (NP_003023.1), and GNTIII (NP_001091740.1) were reverse translated and codon-optimized by Geneart (Life Technologies) and ordered at Geneart (B4GALT2, ST6GALT) or at Integrated DNA technologies (B4GALT1, GNTIII). For sub cloning, restriction sites HindIII and EcoRI were included at the 5′ prime and 3′ prime end of sequence respectively, as well as a Kozak sequence at 5′ prime of the coding sequence prior to the start codon. The plasmid containing the sequence for the RMD gene was described previoulsy^38^. All constructs were subsequently sub cloned into an expression vector using the flanking HindIII and EcoRI restriction sites. B4GALT1, B4GALT2, GNTIII into pEE6.4 (Lonza), ST6galT into pEE14.4 and RMD into pcDNA3.1 (Invitrogen, V790-20). To enhance IgG production, vectors encoding for p21 (Invivogen, porf-hp21), p27 (Invivogen, pORF-hp27 v02), and adenovirus large –T antigen^53^ were used as described by Vink *et al*.^53^.

### Cells and media

For the production of IgG we used the serum-free FreeStyle™ 293 Expression System (Invitrogen). Cells were cultured in 293 Freestyle^®^ expression medium (Invitrogen, 12338018), at 37 °C, 8% CO_2_, on a shaking platform with rotation speed 125 RMP and rotation radius of 25 mm. For large volume cultures 0.125, 0.25, 0.5 or 1 L Erlenmeyer flasks (Corning, 431143, 431144, 431145 or 431147) were used, for small volume cultures non-treated 6-well culture plates were used (Corning, 3736). Cells were maintained as prescribed by Invitrogen. Cell count was measured using CASY cell counter (Roche Innovatis, Reutlingen, Germany). For the transfection of HEK freestyle cells Opti-MEM^®^ (Gibco, Thermo Fisher Scientific, 31985062) and 293-Fectin™ (Invitrogen, Thermo Fisher Scientific, 12347019) was used.

### Transfection of IgG

Further transfection procedure was according to manufacturer’s protocol (Invitrogen) and modified for IgG production as described by Vink *et al*.^53^. Cell supernatant was harvested after 5 days, cells were pelleted by centrifugation at maximum speed (≥4000 g) and supernatant was filtered using a 0.45 nm puradisc syringe filter (Whatmann, GE Healthcare, 10462100). IgG1 concentration in supernatant was determined by enzyme-linked immunosorbent assay (ELISA) as previously described by Kapur *et al*.^54^.

### Glyco-engineering

Where indicated, galactose substrate, D-galactose (Sigma, G0750-5G), was added to the medium before transfection. The decoy substrates for fucosylation, 2-deoxy-2-fluoro-l-fucose (2FF) (Carbosynth, MD06089) and galactosylation 2-deoxy-2-fluoro-d-galactose (2FG) (Carbosynth, MD04718) were added 4 hours post transfection. All carbohydrates were diluted in distilled sterile water and filtered using a 0.2 nm puradisc syringe filter (Whatmann, GE Healthcare, 10462200). Percentages of co-transfection of enzyme (glycosyltransferase or RMD) vectors ware calculated relative to total DNA, keeping the amount of antibody-encoding vector constant, and adding an empty expression vector to keep the amount of total DNA constant. *In vitro* sialylation was performed with recombinant human α-2,6-sialyltransferase (Roche, 07012250103) and cytidine-5′-monophospho-*N*-acetylneuraminic acid (CMP-NANA) (Roche, 05974003103). Antibody, enzyme and substrate were mixed in a 20:1:10 w/w ratio in PBS at pH 7.4, incubated for 12 hours at 37 °C, subsequently additional CMP-NANA was added to the mixture to a final ratio of 20:1:20 for antibody, enzyme and substrate respectively and further incubated for 12 hours at 37 °C. After *in vitro* sialylation to remove the excess enzyme and CMP-NANA, the antibodies were re-purified on a protein A (WT IgG1) HiTrap HP column (GE Life Sciences) as described below.

### Purification of IgG1

Antibodies were purified from the supernatant on a protein A (WT IgG1) HiTrap HP column (GE Life Sciences, 29-0485-76) and dialyzed against phosphate buffered saline (PBS) overnight. IgG concentration was determined by measuring A_280_ on a nanodrop 2000c UV/VIS spectrophotometer (Thermo Scientific, Waltham, MA USA) When produced in small volumes (<25 ml) IgGs were purified using CaptureSelect anti-human Fc agarose beads (CaptureSelect™ IgG-Fc (Hu) Affinity Matrix, Life Technologies, 190082205). 300 μl supernatant was incubated with beads in a ratio of 20:1 v/v in a 96-well filter plate (Orochem, 10 μm polyethylene frit1, OF1100). After 1 hour incubation, beads were washed three times with PBS and three times with milli-Q water (Merck Millipore, MA). IgG was eluted by incubating the beads in 100 mM formic acid (Sigma-Aldrich, 94318). Purified IgG from protein A purification was also dissolved in 100 mM formic acid.

### Digestion of IgG for mass spectrometry

Dissolved IgGs from both methods were immediately dried for 2 hours at 60 °C in a centrifugal vacuum concentrator (Eppendorf, Hamburg, Germany). The samples were resuspended and digestion took place overnight at 37 °C in 25 mM ammonium bicarbonate (Sigma-Aldrich, 09830) with 200 ng trypsin (sequencing grade, Promega, V5111) in a volume of 40 μl.

### Mass spectrometric glycosylation analysis

The trypsin-digested glycopeptide samples were analysed by nanoLC-ESI-QTOF-MS. The separation was performed on an RSLCnano Ultimate 3000 system (Thermofisher, Breda, the Netherlands) with a gradient pump, loading pump and an autosampler. An amount of 250 nl of sample was injected and washed on a Dionex Acclaim PepMap100 C18 trap column (5 mm × 300 μm i.d.; Thermofisher) for 1 min with 0.1% TFA at a flow rate of 25 μl/min. The sample was then separated on an Ascentis Express C18 nanoLC analytic column (50 mm × 75 μm i.d.; 2.7 μm fused core particles; Supelco, Bellefonte, PA) with a flow rate of 0.9 μl/min. The following linear gradient was used, with mobile phase A consisting of 0.1% TFA in milli-Q and mobile phase B of 95% CH3CN and 5% milli-Q: t = 0 min, 3% B; t = 2, 6% B; t = 4.5, 18%B, t = 5, 30%B; t = 7, 30% B; t = 8, 1% B; t = 10.9, 1% B. The observed IgG1 Fc glycopeptides elute in a narrow range and are largely overlapping. While nanoLC with formic acid/acetonitrile separates neutral from acidic species^26^, the use of TFA/acetonitrile results in highly comparable migration of neutral and acidic glycoforms allowing the coverage of all glycoforms in a rather narrow sum spectrum. The resulting co-elution of the different glycoforms of the IgG1 Fc glycosylation site warrants fair comparison by ensuring identical ionization conditions for the various glycopeptide species. The LC was coupled to the MS detector via a CaptiveSpray source with a NanoBooster (Bruker Daltonics, Bremen, Germany). The latter enriched the N_2_ flow (3 L/min) with CH3CN (pressure 0.2bar), resulting in increased sensitivity. The samples were ionized in positive ion mode at 1100 V. The Maxis Impact quadrupole-TOF-MS (micrOTOF-Q, Bruker Daltonics) was used as detector. MS1 spectra were collected at a frequency of 1 Hz with a scan range of *m/z* 550-1800. The mass spectrometric data was calibrated internally in DataAnalysis 4.0 (Bruker Daltonics) using a list of known IgG glycopeptide masses. MSConvert (Proteowizard 3.0)^55^ was used to convert the data files to mzXML format, and an in-house alignment tool^56^ was used to align the retention times of the data files. The highest intensity of selected peaks (within an *m/z* window of +/−0.2 and within a time window of +/−15 s surrounding the retention time) was extracted using the in-house developed 3D Max Xtractor software tool. A list of the glycopeptides which were extracted can be examined in Supplementary Table 1. If above a signal:background ratio of 3, the background-subtracted area of the first 3 isotopic peaks of each glycopeptide in both 2+, 3+ and 4+ charge state were summed, and this summed value was then divided by the total summed value of all IgG1 glycopeptides to arrive at a percentage for each glycopeptide. From these percentages we calculated several derived traits using the following formulas: fucosylation (H3N3F1 + H4N3F1 + H5N3F1 + H6N3F1 + G0F + G1F + G2F + H6N4F1 + G0FN + G1FN + G2FN + H6N5F1 + H4N3F1S1 + H5N3F1S1 + H6N3F1S1 + G1FS + G2FS + H6N4F1S1 + G2FS2 + G1FNS + G2FNS + H6N5F1S1 + G2FNS2), bisection (H6N4F1 + G0FN + G1FN + G2FN + H6N5F1 + H6N4F1S1 + G1FNS + G2FNS + H6N5F1S1 + G2FNS2 + H6N4 + G0N + G1N + G2N + H6N5 + H6N4S1 + G1NS + G2NS + H6N5S1 + G2NS2), galactosylation ((H4N3F1 + H5N3F1 + G1F + H6N4F1 + G1FN + H6N5F1 + H4N3F1S1 + H5N3F1S1 + H6N3F1S1 + G1FS + H6N4F1S1 + G1FNS + H6N5F1S1 + H4N3 + H5N3 + H6N3 + G1 + H6N4 + G1N + H6N5 + H4N3S1 + H5N3S1 + H6N3S1 + G1S + H6N4S1 + G1NS + H6N5S1) * 0.5 + G2F + G2FN + G2FS + G2FS2 + G2FNS + G2FNS2 + G2 + G2N + G2S + G2S2 + G2NS + G2NS2), sialylation ((H4N3F1S1 + H5N3F1S1 + H6N3F1S1 + G1FS + G2FS + H6N4F1S1 + G1FNS + G2FNS + H6N5F1S1 + H4N3S1 + H5N3S1 + H6N3S1 + G1S + G2S + H6N4S1 + G1NS + G2NS + H6N5S1) * 0.5 + G2FS2 + G2FNS2 + G2S2 + G2NS2), hybrid-types (H5N3F1 + H6N3F1 + H6N4F1 + H6N5F1 + H5N3F1S1 + H6N3F1S1 + H6N4F1S1 + H6N5F1S1 + H5N3 + H6N3 + H6N4 + H6N5 + H5N3S1 + H6N3S1 + H6N4S1 + H6N5S1) and high-mannose (H5N2 + H6N2 + H7N2 + H8N2 + H9N2). For some of the minor hybrid-type glycans it could not be determined conclusively whether a galactose or a bisecting *N*-acetylglucosamine was present, so an educated guess was made based on structural knowledge (for instance, since the hybrid glycan H6N4F1 is elevated in GNTIII-co-transfected HEK cell-derived IgG samples, it is likely to be a bisected species rather than triantennary).

### Statistical analysis

Statistical analyses were performed using GraphPad Prism version 6.00 for Windows (GraphPad Software, La Jolla, CA). The level of significance was set at p < 0.05 using two-tailed tests. Distributions for glycan traits for HEK-Freestyle derived cells were found to be normally distributed for multiple samples treated identically (verified by D’Agostino & Pearson Omnibus normality test). A one-way ANOVA using Dunnett’s multiple comparisons tests were used comparing untreated cells with treated, unless otherwise indicated when test Student’s-t-test was used.

## Additional Information

**How to cite this article**: Dekkers, G. *et al*. Multi-level glyco-engineering techniques to generate IgG with defined Fc-glycans. *Sci. Rep.* **6**, 36964; doi: 10.1038/srep36964 (2016).

**Publisher’s note:** Springer Nature remains neutral with regard to jurisdictional claims in published maps and institutional affiliations.

## Supplementary Material

Supplementary Figures and Table 1
