# Supplementary material for: Multi-level glyco-engineering techniques to generate IgG with defined Fc-glycans
Source: Sci Rep. 2016 Nov 22;6:36964. doi: 10.1038/srep36964 (PMC5131652; doi:10.1038/srep36964)
Supplement: Supplementary Figures and Table 1 [file srep36964-s1.pdf]

Supplemental files for:

**Multi -level glyco-engineering techniques to generate IgG with defined Fc-glycans**

Gillian Dekkers, Rosina Plomp, Carolien A.M. Koeleman, Remco Visser, Hans H. von Horsten, Volker Sandig, Theo Rispens, Manfred Wuhrer, and Gestur Vidarsson

# Dekkers et al. Supplemental figure 1

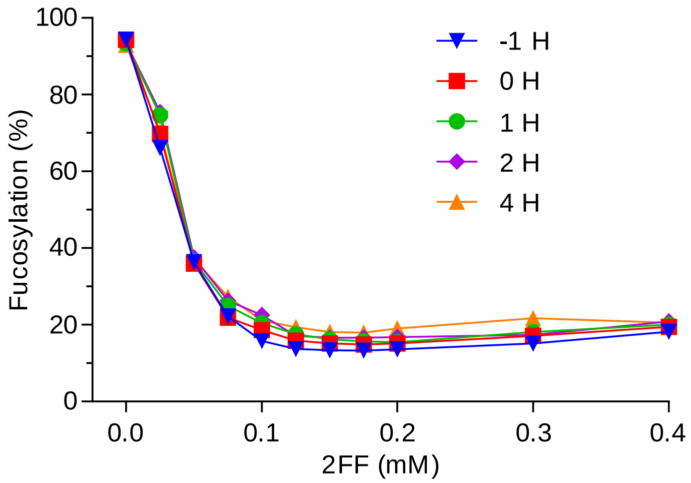


**Fig. S1 Optimization of 2FF addition**

The fucose level of IgG1 N297, produced by transfection of IgG heavy and light chain vector with addition of 2-deoxy-2-fluoro-fucose (2FF) in a concentration range and added at different time points indicated as in hours (H) relative to the time of transfection.

# Dekkers et al. Supplemental figure 2

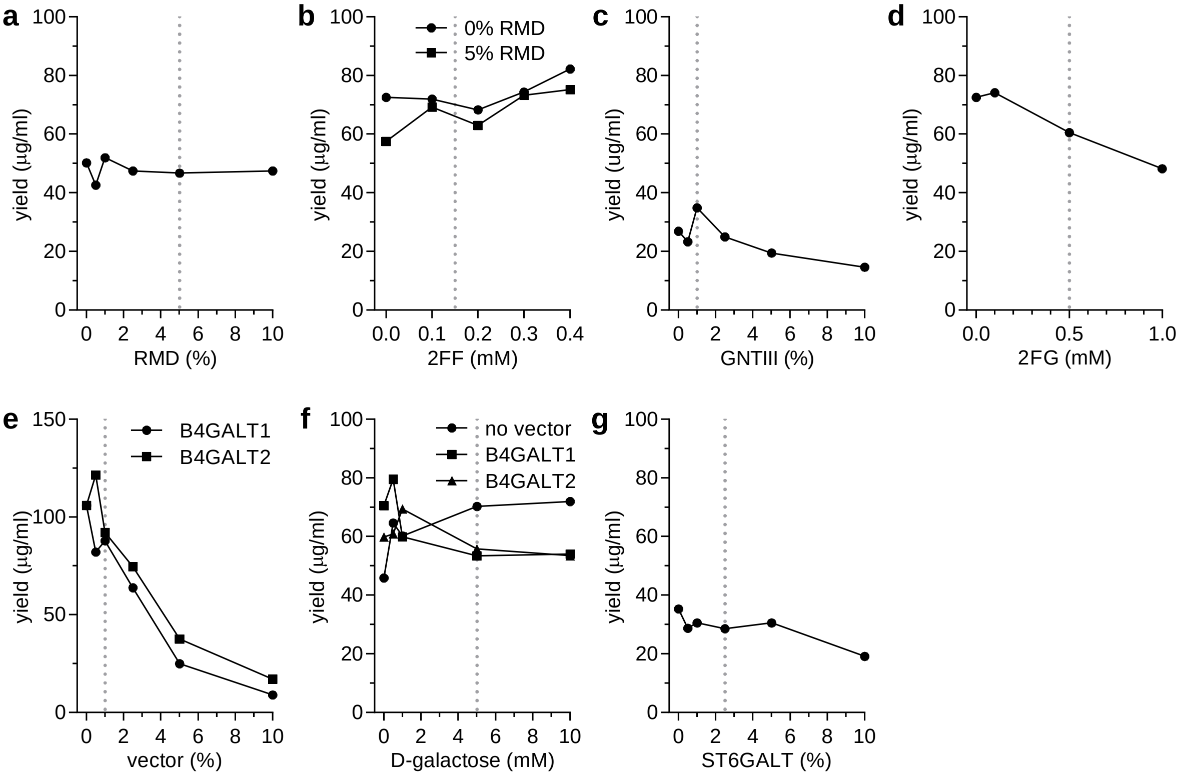


**Fig. S2 Yield of production ranges**

Representative experiments of at least 2 independent experiments of yield of IgG production in different glyco-engineering experiments as determined by IgG1 ELISA, A) RMD co-transfection, B) combination of RMD co-transfection and 2FF addition, C) GNTIII co-transfection, D) 2FG addition, E) B4GALT1 or B4galT2 co-transfection, F) combination of either no, 1% B4GALT1 or 1% B4GALT2 co-transfection with D-galactose addition, G) ST6GALT co-transfection in combination with 1% B4GALT1 co-transfection and 5mM D-galactose addition. The vertical dotted lines in represent the designated optimal concentrations of the indicated vector or substrate.

Dekkers et al. Supplemental figure 3

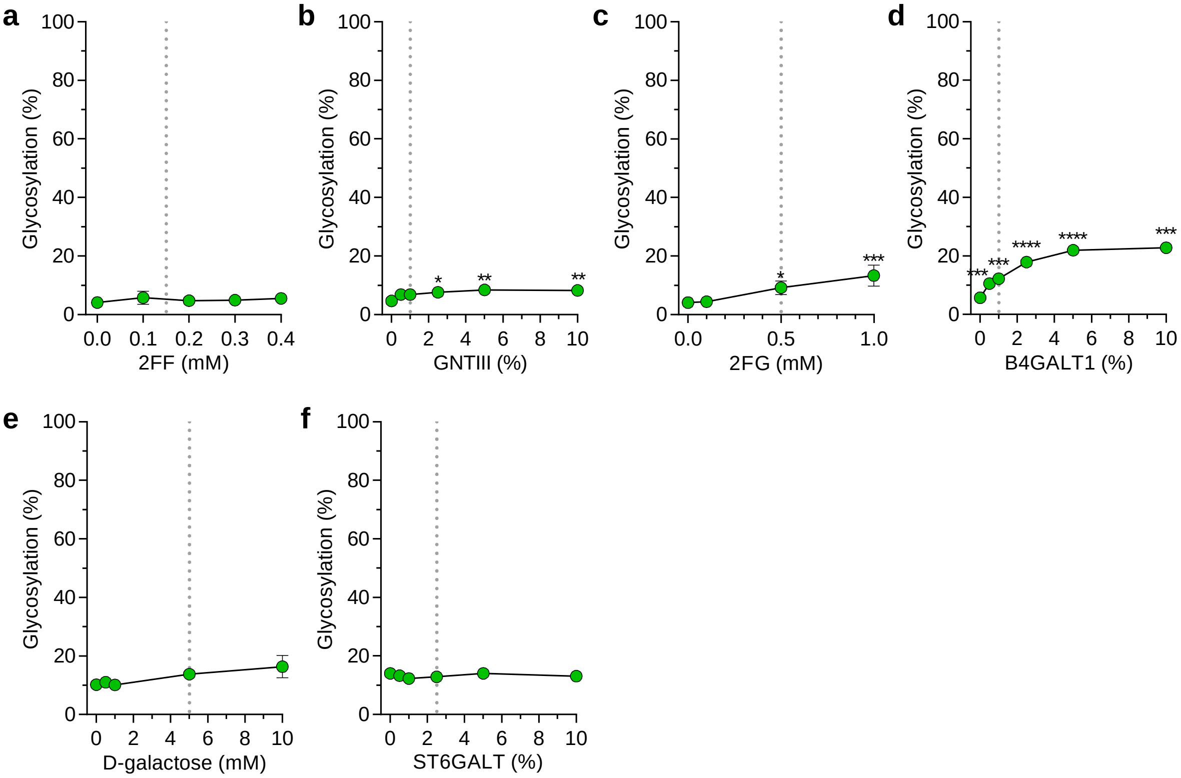


**Fig. S3 High-mannose and hybrid-type glycosylation**
Effect of the different glyco-engineering tools on sum of high-mannose and hybrid-type glycosylation traits; A) 2FF addition (N=3), B) GNTIII co-transfection (N=2), C) 2FG addition (N=4), D) B4GALT1 co-transfection (N=2), E) 1% B4GALT1 co-transfection with D-galactose addition (N=2), F) 1% B4GALT1 co-transfection and 5mM D-galactose addition in combination with ST6GALT co-transfection (N=2). The data represents means and SD combined independent experiments, *, **, *** and **** denote a statistical significance of $p \leq 0.05$, $p \leq 0.01$, $p \leq 0.001$ and $p \leq 0.0001$, respectively, as tested by one-way ANOVA using Dunnett's multiple comparisons test. The vertical dotted lines in represent the designated optimal concentrations of the indicated vector or substrate.

# Dekkers et al. Supplemental table 1

| IgG1 glycan species | | theoretical monoisotopic *m/z* | | |
|---|---|---|---|---|
| **monosacharide composition** | **alternative nomenclature** | **$[M+2H]^{2+}$** | **$[M+3H]^{3+}$** | **$[M+4H]^{4+}$** |
| **H3N3F1** | - | 1215.987 | 810.994 | 608.497 |
| **H4N3F1** | - | 1297.013 | 865.011 | 649.010 |
| **H5N3F1** | - | 1378.040 | 919.029 | 689.523 |
| **H6N3F1** | - | 1459.066 | 973.046 | 730.037 |
| **H3N4F1** | G0F | 1317.527 | 878.687 | 659.267 |
| **H4N4F1** | G1F | 1398.553 | 932.704 | 699.780 |
| **H5N4F1** | G2F | 1479.579 | 986.722 | 740.293 |
| **H6N4F1** | - | 1560.606 | 1040.740 | 780.807 |
| **H3N5F1** | G0FN | 1419.066 | 946.380 | 710.037 |
| **H4N5F1** | G1FN | 1500.093 | 1000.398 | 750.550 |
| **H5N5F1** | G2FN | 1581.119 | 1054.415 | 791.063 |
| **H6N5F1** | - | 1662.145 | 1108.433 | 831.576 |
| **H4N3F1S1** | - | 1442.561 | 962.043 | 721.784 |
| **H5N3F1S1** | - | 1523.587 | 1016.061 | 762.297 |
| **H6N3F1S1** | - | 1604.614 | 1070.078 | 802.811 |
| **H4N4F1S1** | G1FS | 1544.101 | 1029.736 | 772.554 |
| **H5N4F1S1** | G2FS | 1625.127 | 1083.754 | 813.067 |
| **H6N4F1S1** | - | 1706.154 | 1137.771 | 853.580 |
| **H5N4F1S2** | G2FS2 | 1770.675 | 1180.786 | 885.841 |
| **H4N5F1S1** | G1FNS | 1645.640 | 1097.429 | 823.324 |
| **H5N5F1S1** | G2FNS | 1726.667 | 1151.447 | 863.837 |
| **H6N5F1S1** | - | out of range | 1205.465 | 904.350 |
| **H5N5F1S2** | G2FNS2 | out of range | 1248.479 | 936.611 |
| **H3N3** | - | 1142.958 | 762.308 | 571.983 |
| **H4N3** | - | 1223.984 | 816.325 | 612.496 |
| **H5N3** | - | 1305.011 | 870.343 | 653.009 |
| **H6N3** | **-** | 1386.037 | 924.361 | 693.522 |
| **H3N4** | G0 | 1244.498 | 830.001 | 622.752 |
| **H4N4** | G1 | 1325.524 | 884.018 | 663.266 |
| **H5N4** | G2 | 1406.550 | 938.036 | 703.779 |
| **H6N4** | - | 1487.577 | 992.054 | 744.292 |
| **H3N5** | G0N | 1346.037 | 897.694 | 673.522 |
| **H4N5** | G1N | 1427.064 | 951.712 | 714.035 |
| **H5N5** | G2N | 1508.090 | 1005.729 | 754.549 |
| **H6N5** | - | 1589.117 | 1059.747 | 795.062 |

# Dekkers et al. Supplemental table 1 continued

| IgG1 glycan species | | theoretical monoisotopic *m/z* | | |
|---|---|---|---|---|
| **monosacharide composition** | **alternative nomenclature** | **$[M+2H]^{2+}$** | **$[M+3H]^{3+}$** | **$[M+4H]^{4+}$** |
| **H4N3S1** | - | 1369.532 | 913.357 | 685.270 |
| **H5N3S1** | - | 1450.558 | 967.375 | 725.783 |
| **H6N3S1** | - | 1531.585 | 1021.392 | 766.296 |
| **H4N4S1** | G1S | 1471.072 | 981.050 | 736.040 |
| **H5N4S1** | G2S | 1552.098 | 1035.068 | 776.553 |
| **H6N4S1** | - | 1633.125 | 1089.085 | 817.066 |
| **H5N4S2** | G2S2 | 1697.646 | 1132.100 | 849.327 |
| **H4N5S1** | G1NS | 1572.611 | 1048.743 | 786.809 |
| **H5N5S1** | G2NS | 1653.638 | 1102.761 | 827.323 |
| **H6N5S1** | - | 1734.664 | 1156.779 | 867.836 |
| **H5N5S2** | G2NS2 | 1799.186 | 1199.793 | 900.096 |
| **H5N2** | - | 1203.471 | 802.650 | 602.239 |
| **H6N2** | - | 1284.497 | 856.667 | 642.752 |
| **H7N2** | - | 1365.524 | 910.685 | 683.266 |
| **H8N2** | - | 1446.550 | 964.703 | 723.779 |
| **H9N2** | - | 1527.577 | 1018.720 | 764.292 |
| **H0N0F0S0** | no glycan | 595.260 | out of range | out of range |

**Supplemental table 1**
Comprehensive list of the IgG1 *N*-glycans which were detected during data analysis. Glycan structures are described in two ways: based on the number of monosacharide residues of which the glycan is composed (with H = hexose, N = *N*-acetylhexosamine, F = fucose and S = N-acetylneuraminic acid) and using the alternative nomenclature often used to describe polyclonal IgG *N*-glycans (with G = galactose, N = bisecting *N*-acetylglucosamine, F = core fucose and S = *N*-acetylneuraminic acid). The theoretical *m/z* value was calculated for each glycopeptide in various charge states. The label 'out of range' was added if the *m/z* value fell outside of the range of the analysis (*m/z* 500-1800).
